# Modifier Ontologies for frequency, certainty, degree, and coverage phenotype modifier

**DOI:** 10.3897/BDJ.6.e29232

**Published:** 2018-11-28

**Authors:** Lorena Endara, Anne E Thessen, Heather A Cole, Ramona Walls, Georgios Gkoutos, Yujie Cao, Steven S. Chong, Hong Cui

**Affiliations:** 1 University of Florida, Gainesville, United States of America University of Florida Gainesville United States of America; 2 The Ronin Institute for Independent Scholarship, Monclair, NJ, United States of America The Ronin Institute for Independent Scholarship Monclair, NJ United States of America; 3 Science and Technology Branch, Agriculture and Agri-Food Canada, Government of Canada, Ottawa, Canada Science and Technology Branch, Agriculture and Agri-Food Canada, Government of Canada Ottawa Canada; 4 CyVerse, Tucson, United States of America CyVerse Tucson United States of America; 5 College of Medical and Dental Sciences, Institute of Cancer and Genomic Sciences, Centre for Computational Biology, University of Birmingham, Birmingham, United Kingdom College of Medical and Dental Sciences, Institute of Cancer and Genomic Sciences, Centre for Computational Biology, University of Birmingham Birmingham United Kingdom; 6 Institute of Translational Medicine, University Hospitals Birmingham NHS Foundation Trust, B15 2TT, Birmingham, United Kingdom Institute of Translational Medicine, University Hospitals Birmingham NHS Foundation Trust, B15 2TT Birmingham United Kingdom; 7 Center for Studies of Information Resources, Wuhan Universtity, Wuhan, China Center for Studies of Information Resources, Wuhan Universtity Wuhan China; 8 National Center for Ecological Analysis and Synthesis, University of California, Santa Barbara, Santa Barbara, United States of America National Center for Ecological Analysis and Synthesis, University of California, Santa Barbara Santa Barbara United States of America; 9 University of Arizona, Tucson, United States of America University of Arizona Tucson United States of America

**Keywords:** frequency modifiers, certainty modifiers, degree modifiers, coverage modifiers, Modifier Ontology, phenotype modifiers, user warrant, literary warrant, user consensus

## Abstract

*Background: *When phenotypic characters are described in the literature, they may be constrained or clarified with additional information such as the location or degree of expression, these terms are called “modifiers”. With effort underway to convert narrative character descriptions to computable data, ontologies for such modifiers are needed. Such ontologies can also be used to guide term usage in future publications. Spatial and method modifiers are the subjects of ontologies that already have been developed or are under development. In this work, frequency (e.g., rarely, usually), certainty (e.g., probably, definitely), degree (e.g., slightly, extremely), and coverage modifiers (e.g., sparsely, entirely) are collected, reviewed, and used to create two modifier ontologies with different design considerations. The basic goal is to express the sequential relationships within a type of modifiers, for example, usually is more frequent than rarely, in order to allow data annotated with ontology terms to be classified accordingly.

*Method: *Two designs are proposed for the ontology, both using the list pattern: a closed ordered list (i.e., five-bin design) and an open ordered list design. The five-bin design puts the modifier terms into a set of 5 fixed bins with interval object properties, for example, one_level_more/less_frequently_than, where new terms can only be added as synonyms to existing classes. The open list approach starts with 5 bins, but supports the extensibility of the list via ordinal properties, for example, more/less_frequently_than, allowing new terms to be inserted as a new class anywhere in the list. The consequences of the different design decisions are discussed in the paper. CharaParser was used to extract modifiers from plant, ant, and other taxonomic descriptions. After a manual screening, 130 modifier words were selected as the candidate terms for the modifier ontologies. Four curators/experts (three biologists and one information scientist specialized in biosemantics) reviewed and categorized the terms into 20 bins using the Ontology Term Organizer (OTO) (http://biosemantics.arizona.edu/OTO). Inter-curator variations were reviewed and expressed in the final ontologies.

*Results:* Frequency, certainty, degree, and coverage terms with complete agreement among all curators were used as class labels or exact synonyms. Terms with different interpretations were either excluded or included using “broader synonym” or “not recommended” annotation properties. These annotations explicitly allow for the user to be aware of the semantic ambiguity associated with the terms and whether they should be used with caution or avoided. Expert categorization results showed that 16 out of 20 bins contained terms with full agreements, suggesting differentiating the modifiers into 5 levels/bins balances the need to differentiate modifiers and the need for the ontology to reflect user consensus. Two ontologies, developed using the Protege ontology editor, are made available as OWL files and can be downloaded from https://github.com/biosemantics/ontologies.

*Contribution: *We built the first two modifier ontologies following a consensus-based approach with terms commonly used in taxonomic literature. The five-bin ontology has been used in the Explorer of Taxon Concepts web toolkit to compute the similarity between characters extracted from literature to facilitate taxon concepts alignments. The two ontologies will also be used in an ontology-informed authoring tool for taxonomists to facilitate consistency in modifier term usage.

## Introduction

Despite the development and use of sensor technology in biomedical domains and applications, phenotypic character descriptions published in the literature remain an indispensable resource for ecological and systematics research.

Anatomical and quality ontologies have been developed to support the curation workflows that aim to convert narrative phenotypical characters to ontological statements for cross-taxon inferences and computation. Uber-anatomy Ontology (UBERON), Hymenoptera Anatomy Ontology (HAO), and the Plant Ontology (PO) are some examples of anatomical ontologies that contain anatomical structure terms and their relationships (Cooper et al. 2013, Yoder et al. 2010, Mungall et al. 2016). The Phenotypic Quality Ontology (PATO) is a taxon-neutral quality ontology that treats character and character value terms (Gkoutos et al. 2017, Gkoutos et al. 2004). These ontologies are often used by EQ-based approaches, where Entity and Quality are post-composed to create an ontological statement for a character (Gkoutos et al. 2009, Dahdul et al. 2018a). Other phenotype ontologies, such as the Flora Phenotype Ontology or FLOPO (Hoehndorf et al. 2016), have also been developed to include complete characters.

Modifier terms are used widely in phenotypic character descriptions but have not been treated formally in an ontology. Hagedorn (2007) provided a good definition for phenotype character modifiers:

*A modifier is a unit of information that adds detail (or constraints) to the statement to which it is applied. When the modifier information is ignored, the original statement must retain a substantial, albeit more general meaning. A modifier may be applied to statements already modified. Modifiers themselves are constrained by a terminology.*

Further, Hagedorn comprehensively summarized the existing studies and arrived at a modifier taxonomy, consisting of 11 groups of modifiers. In this work, we attempt to construct modifier ontologies that treat four groups of the modifiers that have general usage across many characters and share the same characteristics of having implied order among the terms, for example, *rarely *is less frequent than *often, perhaps *is less certain than *clearly*. This sequential relationship is the key semantics we would like to capture in the modifier ontologies because it will be the key for a computer to understand:

1. How to compare modifiers semantically
2. When to inherit a character from a family level description to a genus level
3. How to use them in an identification key application

We propose two alternative approaches to constructing a modifier ontology and discuss the tradeoffs between the two. Both approaches are grounded to a set of modifier words extracted from 30 volumes of Flora of North America (Flora of North America Editorial Committee 1993), the Flora of China (Flora of China Editorial Committee 1994), and a large number of taxonomic publications (ca. 21,000 treatments) on ants, algal fossils, and other taxon groups.

### Related work

While a standard formula for building ontologies is yet to be proposed, Z39.19 National Standard for Monolingual Controlled Vocabulary Construction NISO (National Information Standards Organization) (2010) Z39.19-2005 laid out the fundamental principles for controlled vocabularies, which apply equally well to ontology building. These principles are “eliminating ambiguity, controlling synonyms, establishing relationships among terms where appropriate, [and] testing and validation of terms” p. 12 of the NISO (National Information Standards Organization) 2010. In addition, the OBO Foundry Principles provide a set of guidelines that OBO Foundry ontologies are expected to follow, covering aspects ranging from ontology content, from definitions and relations (mostly under-development) to ontology management (Smith et al. 2007).

The Basic Formal Ontology (BFO, Arp et al. 2015; https://raw.githubusercontent.com/BFO-ontology/BFO/v2.0/bfo.owl, accessed 4/18/2018) provides a genuine domain independent upper ontology that differentiates a number of fundamental concepts that are useful to guide the development of many ontologies.

Within the BFO framework, character modifiers would fall under the Specifically Dependent Continuant > Quality class. PATO is a taxon-neutral quality ontology (Gkoutos et al. 2004) with the root class “quality” and is tasked to supply quality terms within the BFO framework. Although it is not specified in PATO, the PATO class for quality encompasses terms that would also be subclasses of BFO’s class “specifically dependent continuant”. For consistency with other trait or phenotype ontologies, we place our root class “modifier” as a subclass of PATO quality.

Hagedorn’s dissertation (Hagedorn 2007) comprehensively reviewed then existing data models for descriptive data of organisms, including those used in DELTA and alike, NEXUS, DiversityDescriptions, CBIT Lucid, XPER and alike, Prometheus, and SDD (Lebbe 1984, Maddison et al. 1997, Pullan et al. 2005, Dallwitz et al. 2000, Hagedorn et al. 2006, Hagedorn 2005, CBIT 2007), each has varied support for different types of modifiers. Hagedorn then grouped modifiers into 11 categories:

1. Spatial modifiers (p. 203, also called “location” or “topological” modifiers). These modifiers indicate a location where a character appear. For example, “at the base”.
2. Temporal modifiers (p. 204) indicate a time when a character appears. For example, “when old”.
3. Method modifiers (p. 205) indicate the method that is used to generate or observe a character, for example, “in alcohol”, and “under hand-lens”.
4. Frequency modifiers (p.206) indicate the probability of observing a true statement, for example, “usually”, “occasionally”, and “rarely”.
5. Certainty modifiers (p. 207) indicate the probability of a statement being true, for example, “perhaps”, “probably”, “likely”, and “certainly”.
6. Approximation modifiers (p. 209), a kind of certainty modifier, indicate the degree of inaccuracy of a reported value. For example, “ca.”, “approximately”, “about”, and “roughly”.
7. Modifiers hinting misinterpretation (p. 209) indicate a stated character is the result of misinterpretation. For example, “by misinterpretation”.
8. Negation modifiers (p. 211) indicate a negation of a stated character. For example, “not red”.
9. State modifiers (p. 212) modify the quality, *degree*, emphasis, or *manner*, etc. of a state itself. For example, “very”, “weakly”, and “slightly”.
10. Reliability modifiers (p. 213) indicate the suitability of a character for the purpose of taxon identification.
11. Other modifiers (p. 214).

The modifier taxonomy proposed in Hagedorn (2007) provides the initial framework for our modifier ontologies.

Over the course of the past ten years, many ontology design patterns have been proposed (e.g., Aranguren et al. 2008, Egaña et al. 2008, Presutti et al. 2012). A design pattern is a general, repeatable solution to a commonly occurring problem. Design patterns have been widely used in software engineering for years to develop reusable and maintainable code bases. The list pattern for ontology development is particularly relevant to modifier ontologies (http://ontologydesignpatterns.org/wiki/Submissions:List accessed 5/27/2018) because the order of the terms is the important semantic relationship that needs to be made explicit to support the applications noted above.

## Material and methods

**Define the Scope**

Ontologies concerning Categories 1-3 in Hagedorn’s taxonomy have been developed or are under development, for example, the Biological Spatial Ontology, (BSPO, Dahdul et al. 2014), the Measurement Method Ontology (Shimoyama et al. 2012), and the Experimental Condition Ontology (Shimoyama et al. 2012). Categories 7 and 10 are defined solely for the purpose of taxon identification and consist of a closed set of system defined terms. These categories are out of scope of the modifier ontology, which focuses on groups of modifiers that have general usage across many characters and are sequentially related to one another. The negation modifiers, or Category 8, was also excluded because negations can be handled with the logical NOT operator. Category 9 derives more specific states from a base state and most of such modifiers are character dependent, for example, “dull” can only modify color characters or sharpness of some edges. However, a subset of the state modifiers, degree modifiers, does have general applicability. Based on this analysis, the scope of our modifier ontologies covers Frequency, Certainty, Degree, and Coverage modifiers (defined below). Coverage modifiers were added after reviewing the candidate terms extracted from a wide range of taxonomic descriptions.

***Frequency*:** *the probability of observing a quality*

***Certainty:*** *the probability of a quality being true*

***Degree:*** *the measure or intensity of a quality, ranging from the minimal to extremely intense*

***Coverage:*** *the spatial extent or scope of a quality, ranging from very sparse coverage to complete coverage of an entity.*

**Data Collection**

Following the literary warrant principle ANSI/NISO (National Information Standards Organization) 2010, we intended for the modifier ontology to include modifier terms used in published taxonomic descriptions. CharaParser (Cui 2012), now a part of the Explorer of Taxon Concepts web toolkit (Cui et al. 2016), was used to parse taxonomic descriptions and extract modifiers from a variety of taxonomic publications (https://www.dropbox.com/sh/msnqb0aqjgwlgaw/AAA-jUfSq14vrnM-AgKSjd49a?dl=0), covering ants, diatoms, plants, and fungi. CharaParser markups biological entities, characters, relationships, and modifiers in taxonomic descriptions. A few thousand unique modifier terms/phrases were extracted and after a manual review of these extracted phrases, 130 unique, one-word modifiers within the scope defined above were selected. Multiple-word phrases or expressions were not considered in this work to limit its scope.

**Modeling**

**Figure 1.**
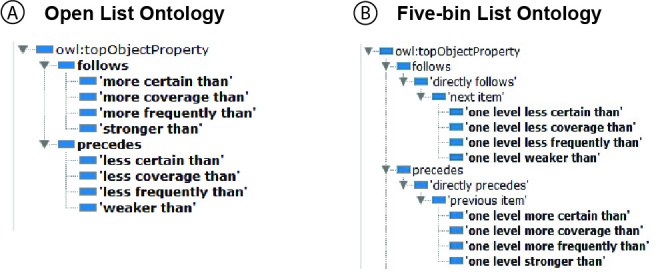
List related object properties in Open List and Five-Bin Ontologies

We observed that the modifier terms were ordinal values. To express the sequential relationships among the terms of each modifier type, two inverse and transitive properties were needed in the ontology: *proceeds* and *follows*. Subproperties of *proceeds* and *follows *can be defined for each of the modifier types, for example, *more_frequently_than and less_frequently_than *(Fig. 1). For some applications, there may be a need to treat these ordinal values as interval values. To support this need, further subproperties can be created, for example, *one_level_more_frequently_than *and *one_level_less_frequently_than,* making the semantic distance between adjacent nodes equal (i.e., “one level”). The form of this set of property and subproperties is similar to the *preceded_by* and *immediately_preceded_by* subproperties of *temporally_related_to* in the Relations Ontology (RO, http://www.obofoundry.org/ontology/ro.html, accessed 5/27/2018), but the former not only takes out the possibility of inserting an intermediate node between two existing nodes, it further equalizes the distances between any adjacent nodes to “one level”. Consumers of the ontology may define the level based on their specific needs.

**Figure 2.**
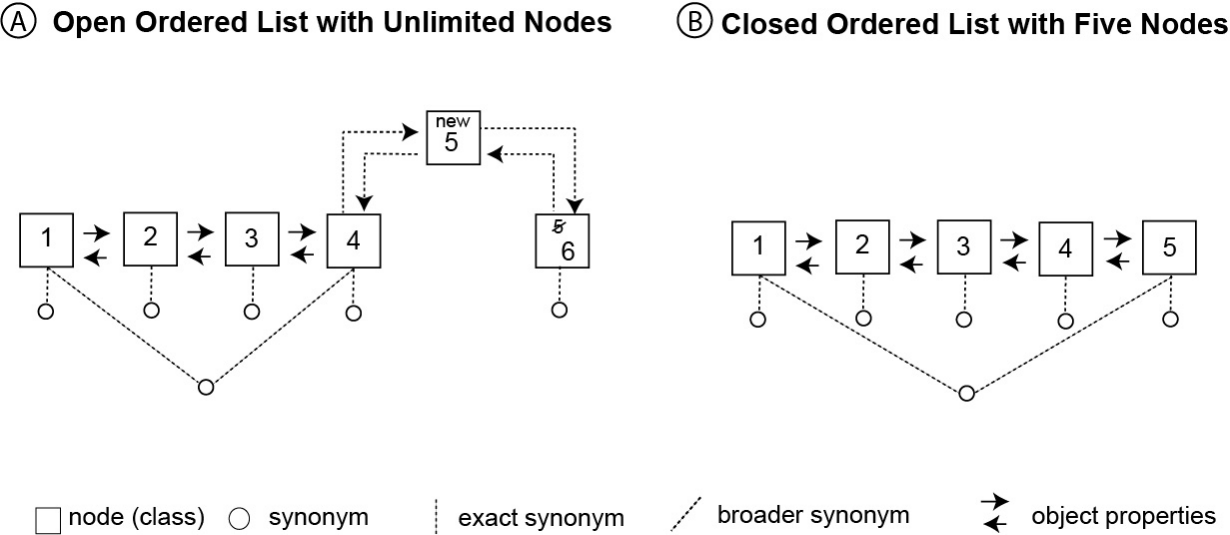
Open List vs. Closed List.

In applying the list pattern to build the modifier ontologies, we have the choice of keeping the list open or making it closed. An ontology was implemented with each of the two approaches. The open list approach does not limit the size of the list (Fig. 2A). Each modifier type is modeled as an open list, where new modifiers can be inserted to the list as classes as long as the* proceeds *and *follows *relationship pairs are established between the new term and their neighboring terms. Fig. 2A shows a conceptual structure of an open list, where a new term (marked as 5) is being inserted into the list.

Similar to the open list approach, in the closed list approach, each modifier type is modeled as a list. However, a closed list has a fixed size, where new modifier terms can only be added as synonyms to some existing nodes (terms) in the list (Fig. 2B).

Open list allows new nodes (i.e., classes) to be inserted anywhere in the list, causing a shift of relative positions of existing nodes, for example, when node 5 is inserted, the original node 5 becomes node 6 (Fig. 2A). Closed list has fixed number of nodes, and new terms can only be added as synonyms. It’s possible for a term to be a synonym of two different nodes, and such a term is a broader synonym of the relevant nodes. Arrowed lines between nodes represent inverse object properties (*proceeds* and *follows*).

Both approaches have desirable and undesirable consequences. An open list is more flexible because not only can new types be easily added as a new list, but new modifier terms can also be added either as a class or a synonym. An open list is not suitable to model interval values because when a new term is added as a class, it changes the positions of all the nodes after the insertion point and therefore the relative positions of affected nodes to all other nodes. This changes the semantic distance between affected nodes. As shown (Fig. 2A), when a new node is added at position 5, the original node 5 becomes node 6, and the distance between this node and node 1 is increased by one. Before the insertion, the similarity between node 5 and node 4 is the same as the similarity between node 4 and node 3. After the insertion, node 5 (now node 6) becomes less similar to node 4 than node 3 is to node 4.

A closed list is a better fit for modeling interval values because the length of the list (the total semantic range) and the position of the nodes in the list are fixed. This fixed structure makes it easy to define the nodes as disjoint classes and to define a list to include only the given classes. This, in effect, creates a “closed world”, making it possible for the machine to classify an unknown entity (i.e., if an unknown entity is one-level preceding node 4 and one-level following node 2, then it must be node 3). Such classification reasoning cannot be done with an open list due to the “open world” assumption of OWL ontologies: the unknown entity may be node 3 but it could also be a node that has not yet been defined.

We also note that open lists allow the ontology to be loaded with more nuanced terms (classes) in a list. Users need to be very cautious when using this feature. Many modifier terms only have subtle differences in meaning and these subtle differences are also quite subjective. This creates two major difficulties in maintaining the ontology’s stability and usability. First, ontology curators and ontology users may not share the same understanding of these terms (and human readable definitions for the terms will not solve this problem). Second, it will be very difficult for different users of the ontologies to use these terms consistently or even for the same users to use these terms consistently over time. The same is true for different curators managing the ontologies.

We implemented two modifier ontologies using the approaches respectively because the need for being flexible and the need for stronger machine reasoning capability seem to be important. Users should decide which implementation better meets their needs.

**Term Categorization Consensus**

Both open and closed list ontologies need to start by crystallizing the sequential relationships among the available terms for a modifier type. To reveal experts’ shared understanding of modifier terms, five bins were created for each of the four modifier types. For example, for the frequency modifiers, the five bins are frequency_0, frequency_25, frequency_50, frequency_75, and frequency_100. The number five was selected to strike a balance between the need to differentiate a good number of levels in each type of modifiers and the requirement for intuitive and consistent categorization of the terms by the users.

**Figure 3.**
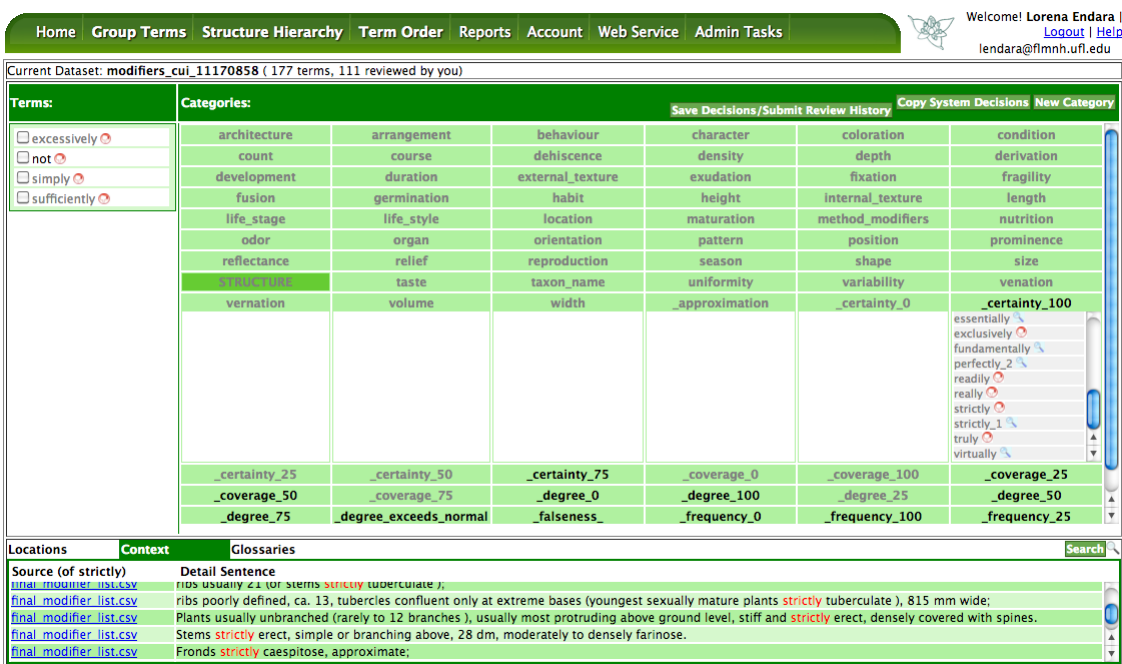
OTO Group Terms User Interface.

The three leading co-authors and the corresponding author categorized the 130 terms into 20 bins (5 bins for each type of modifier) using OTO (Huang et al. 2015, http://biosemantics.arizona.edu:8080/OTO). Since the terms are on the ordinal scale, the experts were not given numerical ranges for the bins but were instructed to simply categorize the terms based on their intuition: do you feel “sometimes” is more similar to 50% frequency or 75% frequency? OTO supports multi-user categorization of terms and synonyms and records all user decisions and comments. It also allows the user to put the same term into multiple bins (Fig. 3). After independent categorization of the terms, experts met virtually and finalized categorization.

Terms to be categorized are in the Terms panel on the left, and the bins are shown in the Categories panel on the right. The source sentences where terms were used are shown in the Context tab in the lower panel. The user drags and drops a term into a bin. The red circle next to a term indicates users have different categorization decisions on the term. Click on the red circle, different decisions will be shown in a pop-up window. Synonyms of a term are shown with an indent below their preferred term. If a term is put into multiple bins, a numerical index is attached to the term to create copies of terms. The term set used in this study is "modifiers_cui_11170858" on OTO, accessible to any OTO registered user.

**Ontology Construction**

After the terms are categorized and categorization reviewed and discussed by the experts, Protege was used to implement the ontologies. Following the user warrant principle (NISO (National Information Standards Organization) 2010), expert consensus on term categorization forms the basis for constructing the ontologies (Tables 1, 2, 3, 4). The following scheme was used to construct a base ontology to which different data properties were then added to create the open list and the five-bin ontologies:

1. Terms with experts’ full agreement on its type and its bin are considered as class label candidates (Table 1).
  1. Within the group of terms for each type and bin (e.g., frequency_75, see Table 1), experts selected one term that best represents the class and this term becomes the class label. This label has the least chance for end users to confuse it with other class labels.
  2. The rest of the terms become the exact synonyms of the class (oboInOWL#hasExactSynonym).
  3. Two exceptions are “throughout” and “uniformly” categorized under coverage_100. This will be discussed in the Discussion section.
2. Terms with experts’ full agreement on its type, but not on its bin are included in the ontology but annotated as “not recommended” (a new annotation), because there is a good chance for the terms to confuse the end users of the ontology. These terms should be included in the ontology as “not recommended” to discourage the continued usage in scientific publications (Table 2).
3. Terms with experts’ full agreement on its bin, but not on its type (Table 3) are included in the ontology as broader synonyms (oboInOWL#hasBroaderSynonym). We follow the best practice of the Plant Ontology Consortium and use broader synonym annotations to indicate if the term is considered a synonym of two or more different classes (Cooper et al. 2013).
4. Terms without full agreement on its type nor its bin are either included as “not-recommended” or excluded from the ontology (Table 4).
  1. Informal terms (colloquial terms) are excluded from the ontology.
  2. If an ambiguous modifier is deemed to have a high probability of being used, it is included in the ontology as a not recommended term.
  3. State modifiers that fell into Category 9 in Hagedorn (2007) were excluded from the ontology as explained in the “Define the Scope” section.
5. For bins where no terms with full agreement is found, experts contributed terms from their vocabulary. Descriptive sentences using these terms were then checked in other sources and terms with full expert agreement were included in the ontology. In Table 1, expert-contributed terms are enclosed with quotation marks.

Classes were given a human readable definition based on their type definition. For example:

Frequently (the class label for Frequency_75) is a *frequency *modifier that indicates around 75% probability of observing a quality.

For the open list ontology, ordinal properties such as *more_frequently_than *and *less_frequently_than* were used to indicate the order of the classes in a list. The five-bin implementation of the ontology uses interval properties such as *one_level_more_frequently_than *and *one_level_less_frequently_than. *In addition, five-bin version also uses *only *(opposed to *some*) existence indicators, disjoint statements, and logical OR operators to make the lists “closed” worlds.

## Results

**Term Categorization Result**

Modifier terms categorized with full agreement on both modifier type and bin accounted for 57.7% of all categorized terms (Table 1). 11.5% terms had agreement on the type, but not on the bin (Table 2), while another 15.4% had agreement on the bin, but not on the type (Table 3). The remainder 16.2% of modifier terms had no agreement on the bin nor the type (Table 4). Four of the twenty bins did not have any terms with full agreement on both type and bin, and three of which are related to coverage. To make the ontology more complete, experts contributed four terms (shown in quotation marks in (Table 1) that filled two of the four empty bins.

**Ontology Result**

**Figure 4.**
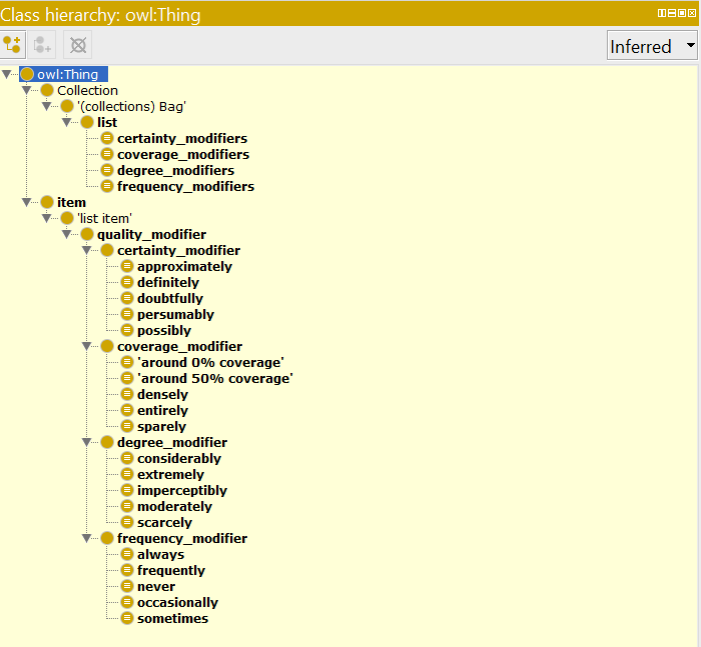
Classes in the modifier ontologies.

Phenotype Modifier Ontology (open list) and Phenotype Modifier Ontology (5-bin) were created, each contains 44 classes and 128 terms. The ontologies can be accessed at https://github.com/biosemantics/ontologies (Fig. 4).

In the current modifier ontologies, a set of inverse object properties are defined for each type of modifier (e.g., *more_frequently_than*, *less_frequently_than* in the open list version, and *one_level_ more_frequently_than*, *one_level_less_frequently_than* in the five-bin version), as opposed to using one generic object property for all types of modifiers (Fig. 1). We believe this treatment better models reality because one level of frequency can be semantically different from one level of certainty. These object properties are subproperties of* follows/precedes *or *next item*/*previous item* properties imported from the list pattern.

## Discussion

An ontology is a conceptual representation of the consensus of a domain. In the modifier domain, we show that there is a level of consensus among the experts: 16 of 20 bins end up holding terms with full agreement. We acknowledge stronger/weaker consensus can be obtained if we had used smaller/larger number of bins. This result suggests that five bins capture a good amount of consensus and a reasonable number of levels most applications need to distinguish within a modifier type. Since the two ontologies share the same set of terms, the consensus gathered from the experts are presented in both. We would like users to decide which ontology works better for their application and it would be interesting to see how the open list ontology evolves with use over time.

In the process of categorizing the terms, Certainty and Degree modifiers were the most difficult to separate among the four types of modifiers. We note that characters that are intense or with great measurements may imply a high certainty of the observation of the character. However, a high certainty does not always correlate with a stronger degree. Based on this observation, terms primarily describing a degree should be categorized as degree and not extended automatically to certainty. For example, authors may have used the words “visibly” and “noticeable” to indicate certainty on characters, however, knowing the ambiguity associated with certainty and degree terms, we need to alert future authors to the difference.

Relatively fewer terms were consistently categorized into Coverage (Table 1). The vast majority (90%) of the terms that had only type disagreement were categorized as Coverage by at least one expert (Table 3). Terms such “mostly” and “generally” are used frequently in phenotype descriptions, but it was not easy to ascertain what the authors tried to express with the term. For example, “leaves *mostly *short-petiolate”, was the author trying to say “leaves *clearly *short-petiolate”(degree), “*most* leaves short-petiolate” (coverage), or even “leaves *usually *short-petiolate” (frequency)? Such terms are included in the ontology with an annotation (broader synonym or not recommended) to alert future authors of the ambiguity with hope that these terms will not be used. We also considered the term “intermittently” as a potential coverage_50 modifier to fill the empty bin in Table 1, but there was only one usage of the term (Table 4) in over 21,000 descriptions included in this exercise, and the experts could not agree on its meaning. We decided to leave the empty bins for future work.

PATO has a frequency class and also treats degree terms to an extent, but they both are different from the modifier ontologies. PATO:frequency (PATO_0000044) is a physical quality of a process, “which inheres in a bearer by virtue of the number of the bearer’s repetitive actions in a particular time”. Based on this definition, PATO:frequency is a quality itself and not a modifier to a quality. Using one example to differentiate the two concepts: a PATO:frequency can be rate of heart beat, say 70 times/min, in contrast, our frequency modifiers describe how often we observe a heart beat of 70 times/min. Hence, frequency modifiers are different from PATO:frequency, conceptually. In our ontologies, we used label “frequency_modifier” to make the difference clear.

PATO employs a consistent pattern of representing the extent of measurable qualities as “decreased”, “increased”, or “normal”, for example, *increased degree of illumination, decreased length*. This is one way to bring out the degree semantics of a quality by referring to an implied normal value. The treatment of degree modifiers in the modifier ontologies is ignorant of any norm, and only attempts to represent the ranges of the degree for a quality.

The concept of modifiers is also used in the Human Phenotype Ontology (Köhler et al. 2016) as reflected in "Clinical modifier" and "Frequency" classes. HPO:Frequency class is similar to our Frequency modifiers in that it bins freqency into a number of ranges: Excluded (0% of the cases), Very rare (1-4%), Occasional (5-29%), Frequent (30-79%), Very frequent (80-99%) and Obligate(100%). HPO:Frequency class is not applicable to our application for several reasons: (1) The class labels (e.g., excluded, obligate) are not terms used by the majority of taxonomists. We believe meaninful class lables are critical to the usability of an ontology. (2) Due to the broad range of various taxon groups we need to cover, precise ranges of percentages of the *cases* are not going to be applicable to all groups. (3) It is very unlikely for various taxon groups to record and compute the percentage of *cases* for an undefined number of characters they may care. HPO:Clinical modifier class holds subclasses "Agravated by", "Ameliorated by", "Pain characteristic", "Phenotpic variability", "Position", "Refractory", "Severity", and "Triggered by". All but "Severity" is disjoint from the types of modifiers that we treat in the modifier ontologies. HPO:Severity overlaps with the Degree modifiers, but it holds subclasses that are applicable to clinical settings: Boderline, Mild, Moderate, Severe, and Profound.

While these ontologies recognize the need to treat modifiers seperately and observed sequential relations among the terms, another key difference between the treatment of modifiers in HPO, as compared to our ontology construct, is that the two Modifier Ontologies we created have clear logic definitions order the terms that form a range, while HPO only has human readable definition. 

The five-bin ontology is currently being used for comparing taxon concepts in the ETC project (Cui et al. 2016). The Taxonomy Comparison tool of the ETC project uses the morphological characters extracted from taxonomic descriptions to facilitate taxon concept resolution tasks. The intuition is that character evidence documented should correlate well with expert asserted relationships between two taxon concepts: if an expert asserts that one taxon concept is congruent with another, then the characters described for the two concepts should be very similar. ETC Text Capture tools extract characters from text for the Taxonomy Comparison tool to compute the similarity between two characters. For example, are “leaves usually toothed” “leaves often toothed”, and “leaves rarely toothed” essentially the same or somewhat different? With an interval list that has a fixed number of elements, as implemented in the five-bin ontology, the software can be configured to reliably compute the similarity score without being affected by ontology updates.

The two ontologies are being applied in another project entitled “Authors in the driver's seat: fast, consistent, computable phenotype data and ontology production”, recently funded by the US National Science Foundation (Cui et al. 2017). Recognizing that the semantic ambiguity in vocabulary usage by the authors at the time of writing results in inconsistent interpretations of documented characters at the time of use (Cui et al. 2015, Endara et al. 2017, Dahdul et al. 2018), the project aims to investigate effective ways to help phenotype authors converge on their term usage and to produce ontology-informed characters for computer algorithms to harvest. These two modifier ontologies will be compared in empirical studies to evaluate their effectiveness for this purpose. For example, the need of authors to add a term as a class vs. a synonym will be examined, in addition to the frequency of authors adopting a modifier from the given classes and exact synonyms.

## Conclusions

The two modifier ontologies were created by following the literary warrant and user warrant principles of the national standard on constructing controlled vocabularies, using the list ontology pattern. The ontologies address four types of modifier terms (frequency, certainty, degree, and coverage) that are used widely in describing phenotype characters but have not been treated by existing ontologies. We have made the ontologies public accessible on GitHub. These ontologies can be used to support machine-based character similarity calculations and to increase author’s awareness of the ambiguities in modifier terms.

## Data resources

Included or linked to within the manuscript

## Figures and Tables

**Table 1.** Frequency, certainty, degree, and coverage modifiers with complete consensus among four experts. Proposed labels are in bold. Expert contributed terms are in quotation marks.

frequency_0	frequency_25	frequency_50	frequency_75	frequency_100
**never**	infrequently, **occasionally**, seldom, uncommonly, rarely	**sometimes**	**frequently**, often, regularly, usually	**always**, consistently
certainty_0	certainty_25	certainty_50	certainty_75	certainty_100
“uncertain” “unclearly” “**doubtfully**”	perhaps, **possibly**	**presumably**, seemingly	**approximately**, nearly	decidedly, **definitely**, distinctly, effectively, essentially, evidentially, evidently, fundamentally,obviously,patently,readily, truly, undoubtedly, virtually
degree_0	degree_25	degree_50	degree_75	degree_100
inconspicuously **imperceptibly** “unnoticeably”	barely, faintly, feebly, gently, hardly, lightly, merely, obscurely, **scarcely, **slightly, subtly	**moderately**, relatively, modestly	appreciably, **considerably**, greatly, highly, much, particularly, profoundly, significantly, strongly, very, noticeably, visibly	boldly, conspicuously, prominently, **extremely **exceedingly**, ** enormously, exceptionally, extraordinarily, grossly
coverage_0	coverage_25	coverage_50	coverage_75	coverage_100
	**sparsely, **sparingly		**“densely”**	**entirely**, throughout, uniformly

**Table 2.** Frequency, certainty, degree, and coverage modifiers with type but not bin consensus among four experts.

**Terms**		Suggested bins		
certainty	almost	certainty_100	certainty_75	
	apparently	certainty_100	certainty_75	
	basically	certainty_100	certainty_75	
	practically	certainty_100	certainty_75	
	probably	certainty_75	certainty_50	certainty_25
	reportedly	certainty_75	certainty_50	
degree	strikingly	degree_100	degree_75	
	notably	degree_50	degree_75	
	quite	degree_50	degree_75	
	rather	degree_50	degree_75	
	fairly	degree_50	degree_25	
	mildly	degree_50	degree_25	
	somewhat	degree_50	degree_25	
	sufficiently	degree_50	degree_100	
	markedly	degree_100	degree_75	

**Table 3.** Terms that have bin consensus but not type consensus among four experts.

Term	Frequency	Degree	Certainty	Coverage
chiefly			_75	_75
mainly			_75	_75
primarily			_75	_75
strictly			_100	_100
exclusively			_100	_100
extensively		_75		_75
fully		_100		_100
totally		_100		_100
completely		_100		_100
largely		_75		_75
mostly		_75		_75
partly		_50		_50
partially		_50		_50
indistinctly		_25	_25	
vaguely		_25	_25	
perfectly		_100	_100	_100
predominantly	_75			_75
prevalently	_75		_75	_75
commonly	_75		_75	_75
typically	_75		_75	_75

**Table 4.** Modifier terms with poor consensus on both type and bin, and their treatment in the ontology

Term	Bins the terms were categorized into by different experts					Treatment of the term for the ontology
	Frequency	Certainty	Degree	Coverage	Other	
altogether			_100		yes	Colloquial, excluded from ontology *E.g., The black spot altogether absent*
casually	_25					State[pattern] modifier, excluded *E.g., Veins regularly or casually anastomosing.*
copiously			_75			State [quantity], excluded from ontology *E.g., Petiole copiously glandular when young*
dominantly	_75		_75, _100	_75		Included as not Recommended *E.g., Cells dominantly solitary, but short chains can be found*
eccentrically					yes	Spatial modifier, excluded *E.g., Anthers eccentrically peltate*
excessively			_75		yes	Not character modifier, excluded *E.g., Females excessively rare*
generally	_75	_50, _75		_75		Included as not Recommended *E.g., head otherwise generally smooth and shining.* *E.g., branches generally quadrangular*
imperfectly			_75	_25		State modifier, excluded *E.g., Rays furcate or imperfectly so.* *Ovary superior, imperfectly 2-loculed*
incompletely			_75			State and other modifier, excluded *E.g., Legumes incompletely 2-locular.* *E.g., Lamina incompletely 2-pinnate at base.* *E.g., Scales incompletely cover underlying leaves.*
intensely			_75, _100		yes	State [color] modifier, excluded *E.g., Petals intensely violet*
intermittently	_50			_25, _50		Included as notRecommended E.g., *Sori spreading intermittently along individual veins almost from midrib to margine.*
no	_0			_0		Negation, excluded
not	_0		_0			Negation, excluded
powerfully			_100			State[Size] modifier, excluded E.g.,* Larvae with mandibles powerfully developed for ant larvae *
really		_100			yes	Not modify characters, excluded E.g., *Really 3 convexities exist.*
remarkably			_75		yes	Included as notRecommended *E.g., Style remarkably exserted. *
richly				_100	yes	Coverage and state modifiers, excluded. *E.g., Vein richly anastomosing* *Stems richly pubescent.*
roughly		_50	_50		yes	State and other modifiers. Included as notRecommended *E.g., Bark roughly furrowed.* *Stigma roughly rectangular.*
simply					yes	State modifier, excluded. *E.g., margin regularly doubly serrate, rarely simply serrate. *
unusually			_75		yes	Included as notRecommended *E.g., Head unusually small*
widely				_100	yes	State modifier, excluded *E.g., Stem leaves widely spaced*
